# Editorial: High added-value nanoparticles: Rethinking and recycling cell protein waste

**DOI:** 10.3389/fbioe.2022.1018014

**Published:** 2022-09-05

**Authors:** Ugutz Unzueta, Julieta M. Sanchez, Elena Garcia-Fruitós, Joaquin Seras-Franzoso

**Affiliations:** ^1^ Biomedical Research Institute Sant Pau (IIB Sant Pau), Barcelona, Spain; ^2^ Josep Carreras Leukaemia Research Institute (IJC, Campus Sant Pau), Barcelona, Spain; ^3^ CIBER de Bioingeniería, Biomateriales y Nanomedicina (CIBER-BBN), Instituto de Salud Carlos III, Madrid, Spain; ^4^ Instituto de Investigaciones Biológicas y Tecnológicas (IIByT), CONICET- Universidad Nacional de Córdoba, ICTA, FCEFyN, UNC, Córdoba, Argentina; ^5^ Department of Ruminant Production, Institute of Agrifood Research and Technology (IRTA), Caldes de Montbui, Spain; ^6^ Drug Delivery and Targeting, Vall d'Hebron Institute of Research (VHIR), Universitat Autònoma de Barcelona, Barcelona, Spain

**Keywords:** nanobiotechnlogy, biomedicine, green biotechnology, Inclusion bodies (IBs), Extracellular vesicles (EVs), virus-based nanomaterials

Nanotechnology, and specifically the use of nanoparticles (NPs), has been intensively explored during the recent decades in order to solve unmet clinical needs and face different biotechnological challenges. However, their success has been rather modest due to several limitations associated with the use of current nanomaterials. In this sense, one of the aspects in which current NPs have failed is in the development of complex formulations, including biological cues in/onto their surface in a cost-effective manner. Moreover, the potential toxicity of the chemicals used for NP synthesis is still an important safety and environmental concern that prevents the implementation of many of such formulations. In this scenario, naturally occurring NPs offer a unique opportunity to overcome these biocompatibility, efficacy and cost-effectiveness issues.

Production of naturally occurring NPs, synthesized either directly within living cells or biofabricated through the application of biological catalyzers, have become an appealing source of NPs with value in the biomedical and biotechnological fields. Such technologies provide unique insights, enabling the preparation of complex nano-sized systems, e.g., Inclusion bodies (IBs) or extracellular vesicles (EVs), formed by a milieu of active biomolecules, in relatively simple ways while rendering highly biocompatible interfaces, optimal for their use in biomedicine. In addition, the diversity in composition and structure of naturally originated NPs empower a significant number of distinct applications also in the biotechnological field, such as their use as immobilized biocatalysts or as biosensors for a spectrum of analytes. This diversity is further fueled by the capacity of tailoring natural NPs structure and functionality in a bottom-up approach via the genetic modification of the biological factories. Among the most common naturally occurring NPs we can find protein-only NPs, IBs, virus-like particles (VLPs), vaults, EVs, magnetosomes or polymeric particles (PP). Besides, a variety of metallic NPs can also benefit from the use of bio-catalyzers during their fabrication. In this regard, the implementation of greener synthesis processes is of paramount importance in the actual context of global environmental crisis.

Concerning the biomedical context, biocompatibility is one of the basic traits that any NP must comply with in order to have a chance to progress towards its clinical use. In this sense, Barguilla et al. validated IBs as sub-micron protein particles displaying no cytotoxicity, genotoxicity or oxidative stress over Caco-2 cells, irrespective of the prokaryotic cell factory employed for their biofabrication. Such reports are very helpful in order to elaborate future product safety profiles and gain insight on the NP/cell interplay. In the same line, but approaching the potential toxicity of NPs from a structural perspective, Gil-Garcia and Ventura reviewed an intriguing strategy to drive IB formation while avoiding the use of potentially toxic amyloid-like domains to pull down proteins into valuable NPs. Thus, standard recombinant manipulation would allow to replace the amyloid (highly beta) conformation of natural IBs by a coiled-coil fold, composed of two or more α-helices. This last protein motif is widely used by nature to create supramolecular assemblies and could reduce potential undesired side effects derived from the amyloid nature of IBs. Nevertheless, numerous studies reported no evident toxicity caused by IBs after challenging cell or animal models (de Marco et al., 2019) and potential IB derived cytotoxicity seems to be linked to specific polypeptide sequences and aggregation status (González-Montalbán et al., 2007).

Of note, the notorious biocompatibility exhibited by IBs is shared by other types of naturally occurring NPs such as EVs. In this regard, EVs are cell membrane bound vesicles secreted by virtually any cell type that can be exploited as efficient delivery systems as exemplified by Dong etal. In this instance, the authors took advantage of EVs properties as natural nanosized drug delivery platforms to envisage an original application for EVs derived from fetal bovine serum (FBS). Serum is a biological fluid extremely rich in EVs and therefore a very interesting source for isolating these NPs in enough amounts. Thus, FBS-derived EVs were functionalized with Icariin as an alternative way to promote osteoblast proliferation and bone regeneration *in vitro*, significantly increasing the observed activity compared to Icariin alone.

With respect to the use of natural NPs in green biotechnology, the present issue also includes diverse and interesting contributions. Shahzadi etal. presented a representative work where spherical silver nanoparticles (AgNPs), with different antioxidant, cytotoxic and antimicrobial activities, were efficiently generated in a cost-effective and environmentally friendly process using plant biotechnology. This is an example of how biological systems can be efficiently used as catalyzers for the biosynthesis of inorganic NPs from externally sourced metal ions, avoiding the use of hazardous chemicals generally employed in the production of such materials. Moreover, AgNPs, biofabricated in this fashion, can additionally incorporate plant-derived phytochemicals, conferring novel antioxidant properties and expanding thus, the spectrum of biological applications of generated nanomaterials.

Another example of green biotechnology is collected here by the virus-based nanomaterials generated by McNulty et al. as reagents for therapeutic protein purification. In this work, virus-based immunosorbent nanoparticles, generated using plant-based manufacturing and virus-functionalized magnetic particles were shown as a potential alternative to the traditional chemical methods used in the biopharmaceutical industry. Of note, such assemblies showed an extraordinary binding affinity capacity for therapeutic proteins.

Finally, a novel usage for bacterial IBs as potential biosensors has been described by Hrabarova etal. This work is yet another example of the enormous potential exhibited by cell protein waste NPs to be re-formulated in order to achieve new objectives. In this regard, the authors successfully engineered a highly soluble green fluorescent protein to produce bioactive IBs with an increased sensitivity to metals and di-/tri-inorganic phosphates. Their approach was based on the addition of small aggregation-prone peptide tags that also show modified sensitivity for poly-phosphates and metals ions. Thus, the same type of biosensors could be used to detect the presence of metal ions such as Cu^2+^, Zn^2+^ or Hg^2+^ in environmental samples or to monitor pyrophosphates levels in plasma, which possess value as a biomarker for various diseases.

All in all, the present research topic reflects the growing interest of natural NPs for different biomedical and biotechnological purposes, where novel applications are steadily appearing and main limitations, e.g., toxicity studies, are being tackled. However, some issues such as: standardization of natural NPs characterization, cumbersome scaling up processes or further understanding of body responses to long term exposure should still be addressed in order to fully fulfill the promise of such NPs.
